# An experimental test of the geodesic rule proposition for the noncyclic geometric phase

**DOI:** 10.1126/sciadv.aay8345

**Published:** 2020-02-28

**Authors:** Zhifan Zhou, Yair Margalit, Samuel Moukouri, Yigal Meir, Ron Folman

**Affiliations:** 1 Department of Physics, Ben-Gurion University of the Negev, Beer-Sheva 84105, Israel.; 2 Research Laboratory of Electronics, MIT-Harvard Center for Ultracold Atoms, Department of Physics, Massachusetts Institute of Technology, Cambridge, MA 02139, USA.

## Abstract

The geometric phase due to the evolution of the Hamiltonian is a central concept in quantum physics and may become advantageous for quantum technology. In noncyclic evolutions, a proposition relates the geometric phase to the area bounded by the phase-space trajectory and the shortest geodesic connecting its end points. The experimental demonstration of this geodesic rule proposition in different systems is of great interest, especially due to the potential use in quantum technology. Here, we report a previously unshown experimental confirmation of the geodesic rule for a noncyclic geometric phase by means of a spatial SU(2) matter-wave interferometer, demonstrating, with high precision, the predicted phase sign change and π jumps. We show the connection between our results and the Pancharatnam phase. Last, we point out that the geodesic rule may be applied to obtain the red shift in general relativity, enabling a new quantum tool to measure gravity.

## Teaser

This paper describes geometric effects in the evolution of atoms through a double-slit type experiment on an atom chip.

## INTRODUCTION

The geometric phase (GP), the phase acquired over the course of an evolution of the Hamiltonian in parameter space, is a central concept in classical and quantum physics (*1*–*9*). Originally, the GP was defined only for an evolution of a system in a closed trajectory in phase space, but later, it was generalized to noncyclic evolutions (*7*, *10*). For the case of a two-level system, where the evolution of the system can be described by a trajectory on the Bloch or Poincaré spheres, it has been proposed (*7*, *10*) that, using a natural definition of the phase (*1*), the GP is given by half the area enclosed by the trajectory and the geodesic connecting the initial and final points. A marked outcome of the proposed geodesic rule is that this noncyclic phase changes sign when the trajectory moves from the upper to the lower hemisphere, resulting in a π-phase jump when the trajectory is half the circumference of a circle (*7*, *10*). While the GP for a closed trajectory has been measured experimentally in several physical systems in a fairly straightforward manner (*11*–*15*), the experimental verification of the GP during noncyclic evolution requires a more convoluted approach. This is so because the cyclic GP can be readily measured as the probed state is returned to its initial position in parameter space, where it can be compared with a reference state to measure the relative phase, while for noncyclic geometric evolutions, where the probed state is not returned to its initial position, one needs to project the final state onto the initial state. Using an ultracold atom spatial interferometer, we test the geodesic rule, including the predicted SU(2) phase sign change and π jumps.

Berry’s original work (*2*) addressed a quantum system undergoing a cyclic evolution under the action of a time-dependent Hamiltonian. When the Hamiltonian returns to its initial value, the quantum state acquires an extra GP in addition to the dynamical phase. This concept has been generalized (*7*) to a noncyclic evolution of the system, where the parameters of the Hamiltonian do not return to their initial values. In addition to the fundamental interest in better understanding the noncyclic behavior, it may also prove to be technologically advantageous. For example, as the system does not need to return to its original state, geometric operations may be done faster, e.g., geometric quantum gates (*16*–*18*). Quantum optimal control of the evolution may also benefit (*19*, *20*). In addition, metrology may be made more sensitive due to the expected phase sign change and phase jumps, e.g., in measuring a gravitational potential (*21*).

**Fig. 1:**
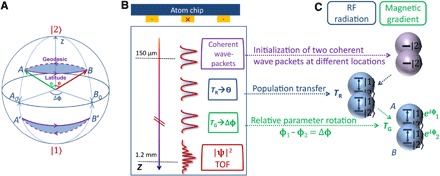
Illustration of the geodesic rule and the experimental sequence. (**A**) An illustration of the geodesic rule (*7*, *10*) on the Bloch sphere representing the two-dimensional space defined by our physical two-level system. The green and red arrows represent the internal states *A* and *B* of the two spatially separated wave packets, Ψ*_A_* and Ψ*_B_* (see Eq. 1). The rotation angle from the north pole θ and the rotation Δϕ along the latitude (continuous purple) represent the SU(2) operations applied in the experiment, where the former requires an RF pulse, while the latter requires a magnetic gradient. When θ = π/2, the arrows lie on the equator of the Bloch sphere (*A*_0_ and *B*_0_). The dashed purple curve is the geodesic joining points *A* (*A*′) and *B* (*B*′). The GP is equal to one-half of the blue area enclosed by the latitude and geodesic. The area’s orientation (indicated by the arrows) is determined by the geodesic rule. It is negative, counterclockwise (northern hemisphere) and positive, clockwise (southern hemisphere). (**B**) Experimental sequence (not to scale) of the longitudinal interferometer. The experiment is performed in free fall. The final interference pattern (from which the total phase is obtained) develops after time-of-flight (TOF) free evolution, in which the two wave packets expand and overlap. The pattern is then recorded by a CCD camera. (**C**) Evolution of the states during the sequence. After the preparation of two coherent wave packets at different locations, an RF pulse of duration *T*_R_ is applied to manipulate θ, and a magnetic field gradient of duration *T*_G_ is applied to manipulate Δϕ.

The geometric interpretation of this noncyclic GP takes an illuminative form for a two-level system whose state can be described by two angles, $\Psi =(\text{cos}\frac{\theta }{2}|2\rangle +\text{exp}\;(i\phi )\text{sin}\;\frac{\theta }{2}|1\rangle )$, which define a point on the Poincaré or Bloch spheres. The propagation of a state under a noncyclic evolution of the Hamiltonian, from Ψ*_A_* to Ψ*_B_*, characterized by {θ*_A_*, ϕ*_A_*} and {θ*_B_*, ϕ*_B_*}, respectively, is represented by a curve connecting points *A* and *B* on the sphere. Using a natural definition of the phase (*1*), where the relative phase between two arbitrary states is zero when the visibility of their interference pattern is maximal, the GP associated with this propagation is determined by the geodesic rule: it is given by half the area on the sphere bordered by the evolution curve and the shortest geodesic connecting *A* and *B* (*22*). An illustration of the geodesic rule on the Bloch sphere is shown in Fig. 1, where *A* evolves toward *B*, along the latitude of fixed θ*_A_* = θ*_B_* = θ, and ϕ changes from ϕ*_A_* to ϕ*_B_* = ϕ*_A_* + Δϕ (the curve $C_{\mathrm{AB}}$). The area corresponding to the GP, blue shaded in the figure, is enclosed by $C_{\mathrm{AB}}$ and by the geodesic curve $G_{\mathrm{AB}}$ joining points *A* and *B*. If $C_{\mathrm{AB}}$ is on the northern hemisphere, $G_{\mathrm{AB}}$ is above (toward the north pole) $C_{\mathrm{AB}}$. But if $C_{\mathrm{AB}}$ is on the southern hemisphere, $G_{\mathrm{AB}}$ is below $C_{\mathrm{AB}}$, leading to a sign change of the GP as $C_{\mathrm{AB}}$ crosses the equator.

Since the introduction of the geodesic rule, several studies have verified it experimentally with light (*23*, *24*), neutron (*25*, *26*), and atom (*27*, *28*) interferometers [see also (*29*) and (*30*) for relevant debates and (*31*) for other interpretations]. In this work, we propose and realize a matter-wave experimental study using cold-atom spatial interferometry (*32*, *33*). The uniqueness of our approach includes (i) the use of a spatial interference pattern to determine the phase in a single experimental run (no need to scan any parameter to obtain the phase), (ii) the use of a common phase reference for both hemispheres and a scan of θ enabling to verify the π phase jump and the sign change, and (iii) obtaining the relative phase by allowing Ψ*_A_* and Ψ*_B_* to expand in free flight and overlap, different from previous atom-interferometry studies that required, for obtaining interference, an additional manipulation of the SU(2) parameters θ and Δϕ. As a result of our novel technique, we are able to test and confirm the geodesic rule for noncyclic evolutions in a new way, including the predicted sign change and the predicted SU(2) phase jumps.

## EXPERIMENT

Our full experimental procedure is detailed elsewhere (*34*–*36*) as well as in Methods and the Supplementary Materials. The relevant part for the determination of the GP is sketched in Fig. 1. The ^87^Rb atom can be in either state ∣1⟩ ≡ ∣ *F* = 2, *m_F_* = 1⟩ or ∣2⟩ ≡ ∣ *F* = 2, *m_F_* = 2⟩, where *F* is the total angular momentum and *m_F_* is the projection. We start by preparing two-atom wave packets at different positions, both in an internal state ∣2⟩. We first apply a uniform radio-frequency (RF) pulse, of time duration *T_R_*, which transfers population from the ∣2⟩ state to ∣1⟩, shifting both wave packets from the north pole of the Bloch sphere to a point whose latitude θ depends on *T*_R_ (Fig. 1A). We then apply a magnetic field gradient pulse of duration *T*_G_, which results, due to the different magnetic moments of states ∣1⟩ and ∣2⟩, in a phase difference between these states, rotating both superpositions along a constant latitude on the Bloch sphere. Because of the difference in positions, each wave packet experiences a different magnetic field and thus will rotate by a different angle, ending up at points *A* and *B* in Fig. 1A. The two states, after the application of both *T*_R_ and *T*_G_, can thus be written as

$$\begin{array}{c} \Psi_{A}=\psi_{A}(r)(\text{cos}\;\frac{\theta }{2}|2\rangle +\text{sin}\;\frac{\theta }{2}|1\rangle )\\ \Psi_{B}=\psi_{B}(r)(\text{cos}\;\frac{\theta }{2}|2\rangle +\text{exp}\;(i\Delta \phi )\text{sin}\;\frac{\theta }{2}|1\rangle ) \end{array}$$ (1)

where θ is proportional to *T*_R_, and Δϕ to *T*_G_. ψ*_A_*(*r*) and ψ*_B_*(*r*) are the spatial components of the respective states. There may also be an additional global phase, identical for both Ψ*_A_* and Ψ*_B_*, which plays no role in the interference between Ψ*_A_* and Ψ*_B_*. To measure this interference, we allow enough time of flight for the two wave packets to free fall, expand, and overlap, before taking a picture using a charge-coupled device (CCD) camera.

## RESULTS

**Fig. 2:**
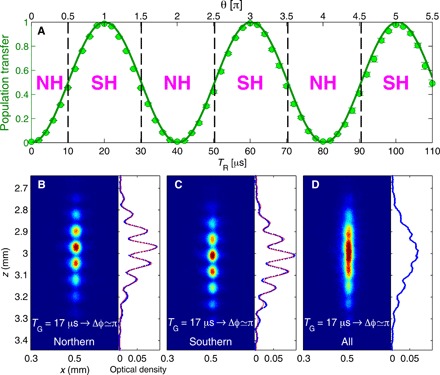
Population transfer and its connection to the π phase jump. (**A**) Population transfer to state ∣1⟩ versus the duration of the RF radiation pulse *T*_R_, for which 20 μs corresponds to total population transfer (θ = π in Fig. 1). With this independent measurement, we determine θ for our SU(2) operations. (**B**) Averaged CCD image of interference when the Bloch vectors are all in the northern hemisphere [NH data points specified in (A)], with Δϕ ≃ π. The high visibility indicates the existence of phase rigidity, namely, that the phase is independent of θ. The phase returned by the fit is 1.13 **±**0.02 rad relative to a fixed reference point, and the visibility is 0.55 ± 0.01 (see Methods for the definition). (**C**) Averaged picture of the second half of the data, in which the Bloch vectors are all pointing in the southern hemisphere [SH data points specified in (A)], with Δϕ ≃ π. A phase jump is clearly visible. The phase is 4.34 **±** 0.03 rad relative to the fixed reference point, which is common to both pictures, and the visibility is 0.52 **±** 0.01. The phase difference between (B) and (C) is thus 3.21 **±** 0.05 rad, close to π. The data included in these images (in total, about 330 consecutive experimental shots without post-selection or post-correction) are presented in Fig. 3B. (**D**) Averaged picture of all the data for Δϕ ≃ π. The visibility is 0.03 **±** 0.01. The low visibility shows that the phase jump has a value close to π. Single-shot data are presented in Fig. 3B, and single-shot images are presented in Fig. 6.

Figure 2 depicts the averaged interference patterns (raw data CCD images) averaged over all values of θ in the upper (B) or lower (C) hemispheres, for *T*_G_ = 17 μs (Δϕ ≃ π). The value of θ was independently deduced from the relative populations of states ∣1⟩ and ∣2⟩, which are given by ${\text{cos}}^{2}(\theta /2)$ and ${\text{sin}}^{2}(\theta /2)$, respectively (Figs. 2A and 5). The high visibility in both images indicates the existence of “phase rigidity,” namely, that the measured phase is independent of θ in each hemisphere. Moreover, the two datasets have a phase difference of π, which can also be deduced from the vanishing visibility in Fig. 2D, where the two datasets in (B) and (C) are joined. Evidently, there is a sharp jump in the phase of the interference pattern as θ crosses the equator.

**Fig. 3:**
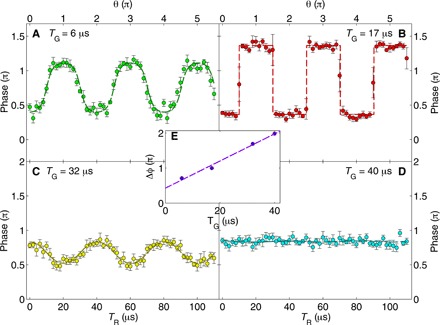
The phase of the interference pattern: Phase jump and rigidity. (**A** to **D**) Total phase Φ as a function of *T*_R_ (θ) for *T*_G_ is equal to 6, 17, 32, and 40 μs. Each data point is an average of six experimental cycles (errors are SEM). The dashed lines are a fit to Eq. 2, which allows us to determine Δϕ for our SU(2) operations. The fit returns the values Δϕ = 2.24 (A), Δϕ = 3.14 (B), Δϕ = 5.31 ≡ 2π − 0.97 (C), and Δϕ = 6.23 ≡ 2π − 0.05 (D) radians, respectively (manifested in the graph as the peak-to-valley amplitude if we consider the periodicity of 2π when defining a phase). The fit also returns a baseline phase ϕ_0_. Last, the phase rigidity and the phase jump observed in Fig. 2 are clearly visible in (B). (**E**) Linear mapping from *T*_G_ to Δϕ. As seen in the graph (*T*_G_ = 0), we have a fixed background gradient equivalent to Δϕ = 1.35.

According to Eq. 1, the interference phase Φ, for general θ and Δϕ, is given by

$$\Phi =\text{arg}\;\;\langle \Psi_{A}|\Psi_{B}\rangle =\phi_{0}+\text{arctan}\;\{\frac{{\text{sin}}^{2}(\theta /2)\text{sin}\;\Delta \phi }{{\text{cos}}^{2}(\theta /2)+{\text{sin}}^{2}(\theta /2)\text{cos}\;\Delta \phi }\}$$ (2)

where ϕ_0_ = arg ⟨ψ*_A_*(*r*) ∣ ψ*_B_*(*r*)⟩ is the phase associated with the evolution of the external degrees of freedom of the system (see Methods). Figure 3 depicts the interference phase, deduced from the raw data, as a function of *T*_R_ for different values of *T*_G_. The dashed lines in this figure are a fit to Eq. 2, with the fitting parameters ϕ_0_ (an overall vertical shift) and Δϕ. The excellent fit to the data allows us to determine with high precision the values of Δϕ (Fig. 3E).

The total phase (interference phase) Φ is a sum of two contributions, the GP Φ_G_ and the dynamical phase Φ_D_. While both Φ and Φ_D_ are gauge dependent, Φ_G_ = Φ − Φ_D_ is gauge independent (*37*, *38*). Substituting for the dynamical phase (*6*, *10*, *37*, *38*), we obtain (see Methods)

$$\Phi_{G}=\text{arctan}\;\{\frac{{\text{sin}}^{2}(\theta /2)\text{sin}\;\Delta \phi }{{\text{cos}}^{2}(\theta /2)+{\text{sin}}^{2}(\theta /2)\text{cos}\;\Delta \phi }\}-\frac{\Delta \phi }{2}(1-\text{cos}\;\theta )$$ (3)

where the gauge-dependent phase ϕ_0_ has dropped out.

**Fig. 4:**
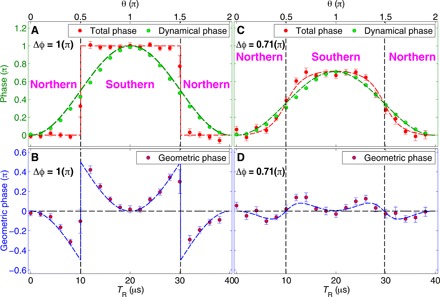
Geometric SU(2) phase jump and sign flip, experiment (dots) versus theory (Eq. 3, dashed lines). (**A**) Total phase and dynamical phase for Δϕ = π as a function of *T*_R_ (θ). The total phase is directly measured from the imaged interference pattern (Fig. 3), and the dynamical phase $\frac{\Delta \phi }{2}(1-\text{cos}\theta )$ is deduced from the independently measured values of θ and Δϕ. (**B**) GP Φ_G_ determined as the difference between the two sets of points appearing in (A). The predicted sign change as the latitude crosses the equator is clearly visible. The evident phase jump is due to the geodesic rule. When Δϕ = π, the geodesic must go through the Bloch sphere pole for any θ ≠ π/2. As the latitude approaches the equator (i.e., increasing θ), the blue area in Fig. 1 (twice Φ_G_) continuously grows to reach a maximum of π in the limit of θ = π/2. As the latitude crosses the equator, the geodesic jumps from one pole to the other pole, resulting in an instantaneous change of sign of this large area and a phase jump of π. This plot exactly confirms the prediction in (*10*). (**C**) Total phase and dynamical phase for Δϕ = 2.24 rad (0.71π). (**D**) Φ_G_, determined as the difference between the two sets of points appearing in (C). The predicted sign change is again visible. However, in the case of Δϕ = 2.24 rad (0.71π), the geodesic line does not go through the pole, and as the latitude approaches the equator, Φ_G_ actually reduces (after reaching its maximum for an intermediate θ), so no abrupt phase jump is expected.

Figure 4 displays Φ, Φ_D_, and the resulting Φ_G_, for two values of Δϕ, where the first term on the right-hand-side of Eq. 3 is given by Φ, the phase of the interference pattern, while the second is evaluated for the experimentally determined values of θ and Δϕ. The dashed lines in Fig. 4 (B and D) correspond to the geodesic rule—half the area between the geodesic and the trajectory, with the correct sign. A very good agreement between data and the theoretical predictions is observed. This constitutes a complete verification of the GP associated with noncyclic evolution in an SU(2) system and accurately confirms the theoretical predictions, including a precise observation of the geodesic rule, the phase sign change, and the π phase jump.

## DISCUSSION

Last, we make a fundamental connection between our experiment and the Pancharatnam phase (*1*). We begin by noting that in the case ϕ_0_ = 0, we have arg ⟨Ψ*_A_* ∣ 2⟩ = 0 and arg ⟨2 ∣ Ψ*_B_*⟩ = 0, and then the states *A*, ∣2⟩, and *B* fulfill the Pancharatnam consecutive in-phase criterion (*1*, *22*). It then follows that arg ⟨*A* ∣ *B*⟩ is given by half the area σ of the spherical triangle defined by these three states on the Bloch sphere, namely, the area in between three geodesic lines. The area σ of the spherical triangle defined by the two arcs joining the north pole and points *A* and *B* is given by the relation $\text{tan}(\sigma /2)=\overset{2}{\text{tan}}(\theta /2)\text{sin}\;(\phi )/[1+\overset{2}{\text{tan}}(\theta /2)\text{cos}\;\phi ]$, which is identical to Eq. 2 with Φ = σ/2 (for ϕ_0_ = 0). This geometric interpretation of Φ yields an explanation of the observed phase rigidity for Δϕ = π: When the two points are in the northern hemisphere, the geodesic between the two points goes through the north pole. The enclosed area is zero; hence, Φ = 0. When the two points are in the southern hemisphere, the geodesic goes through the south pole, with an area of 2π, resulting in a jump of π in the value of Φ (Fig. 2). The geometric interpretation of our experiment is now evident, namely, what is measured in the experiment (the interference-pattern phase) is the Pancharatnam phase Φ_P_. The difference between the areas associated with Φ_P_ and Φ_D_ gives the light blue area in Fig. 1, associated with Φ_G_. This now naturally explains both the sign change of Φ_G_ as the latitude crosses the equator, as well as the phase jump for Δϕ = π (Fig. 4).

## OUTLOOK

As an outlook, we consider a situation in which the two wave packets are viewed as a split wave packet of a single clock, where θ = π/2 for a perfect two-level clock (*35*, *36*). When we place the two wave packets along a vertical line parallel to gravity at different distances from earth, they are exposed to different proper times. In the experiment described in this paper, the relative phase Δϕ = Δ(*E*_1_ − *E*_2_) × *t*/ħ between the wave packets is determined by a magnetic gradient, which changes the energy splitting *E*_1_ − *E*_2_ between states ∣1⟩ and ∣2⟩ [i.e., Δ(*E*_1_ − *E*_2_) is the difference of energy splitting between two wave packets Ψ*_A_* and Ψ*_B_*], while time (from the moment the two wave packets were allowed to free fall) is the same for both wave packets. However, the same GP situation occurs when the magnetic gradient is zero and consequently the splitting *E*_1_ − *E*_2_ is identical for the two wave packets, but time elapsed is different for the two wave packets due to the different red shift (with time difference Δ*t*). In this case, we have Δϕ = (*E*_1_ − *E*_2_) × Δ*t*/ħ, and the same theory presented in this paper may be used to analyze via the GP an experimental situation on the interface between quantum mechanics and general relativity. Moreover, by scanning θ around π/2 (i.e., change the relative populations of the ∣1⟩ and ∣2⟩ states from below to above half), one should observe a sign change that may allow the construction of a novel type of gravitational sensor. A main limitation that will have to be examined is the sharpness of the slope. Even when working at the best point (ΔΦ = π; see Fig. 4B), the practical slope will never be the theoretical infinite slope, as the visibility at this exact point is zero (as may be seen from the large error bar). However, the visibility quickly recovers and numerical and experimental studies are needed to reveal the ultimate realizable slope. An additional limitation has to do with the fact that the sharp slope appears when Δϕ = π, so when dealing with proper time differences that give rise to a substantially different Δϕ, a bias would have to be introduced to keep the system at this optimal point, and this bias may introduce its own errors.

## METHODS

### Detailed experimental scheme

The experiment was realized in an atom chip setup (*39*). We present the detailed experimental scheme in fig. S1, which includes the two-level system preparation. We first prepared a Bose-Einstein condensate (BEC) of about 10^4 87^Rb atoms in the state ∣2⟩ ≡ ∣ *F* = 2, *m_F_* = 2⟩ in a magnetic trap located 90 μm below the chip surface. After the BEC atoms were released from the trap, the entire experimental sequence took place in the presence of a homogeneous magnetic bias field of 36.7 G in the *y* direction (*z* is the direction of gravity), which created an effective two-level system (with ∣1⟩ ≡ ∣ *F* = 2, *m_F_* = 1⟩) via the nonlinear Zeeman effect with *E_ij_* = *E*_21_ ≈ *h* × 25 MHz (where *i* and *j* are the *m_F_* numbers, all in the *F* = 2 manifold), and *E*_21_ − *E*_10_ ≈ *h* × 180 kHz. We then applied an RF pulse (duration *TR*_1_, where typically 10 μs gives rise to a θ = π/2 rotation) to prepare a spin superposition $(|1\rangle +|2\rangle )/\sqrt{2}$ between the ∣2⟩ and ∣1⟩ states. A magnetic gradient pulse ∂*B*/∂*z* of duration *TG*_1_ = 4 μs, generated by currents in the atom chip wires, was applied to create the Stern-Gerlach splitting, in which the different spins are exposed to differential forces. To enable interference between the two wave packets (∣2⟩ and ∣1⟩ are orthogonal), a second π/2 pulse (TR_2_) was applied to mix the spins. To stop the relative velocity of the wave packets, a second magnetic gradient pulse (TG_2_) was applied to yield differential forces for the same-spin states at different locations. A spatial superposition of two wave packets in state ∣2⟩ now exists (separated along the *z* axis, with zero relative velocity). Note that during TG_2_, the ∣1⟩ state from the two wave packets was pushed outside the experimental zone. The control of θ introduced in Fig. 1A is realized by a third RF pulse of duration TR_3_ (*T*_R_ in the main text). The relative rotation between the two wave packets Δϕ may be changed by applying a third magnetic field gradient of duration TG_3_ (*T*_G_ in the main text). The wave packets were then allowed to expand (during time of flight of ∼10 ms, much larger than the reciprocal of the trap frequency ∼500 Hz) and overlap to form the interference pattern. An image based on the absorption imaging was taken in the end.

The magnetic gradient pulses were generated by three parallel gold wires located on the chip surface with a length of 10 mm, a width of 40 μm, and a thickness of 2 μm. The chip wire current was driven using a simple 12.5-V battery and modulated using a homemade current shutter. The three parallel gold wires were separated by 100 μm (center to center), and the same current runs through them in alternating directions. The benefit of using this three-wire configuration instead of a single gold wire is that a two-dimensional quadrupole field was created at *z* = 100 μm below the atom chip. As the magnetic instability is proportional to the field strength, and as the main instability originates in the gradient pulses (the bias fields from external coils are very stable), positioning the atoms near the middle (zero) of the quadrupole field significantly reduces the magnetic noise while maintaining the strength of the magnetic gradients.

### Determination of the population transfer and the value of θ

**Fig. 5:**
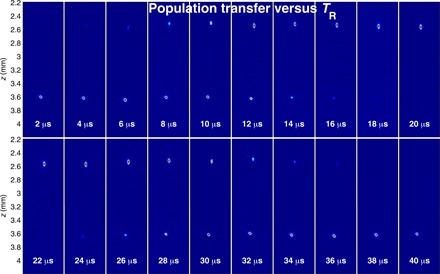
The population transfer versus *T*_R_ is measured in an independent experiment by applying a strong magnetic gradient after *T*_R_. Because of the Stern-Gerlach effect, the *m_F_* = 1 and *m_F_* = 2 parts are shifted to different regions of space when the absorption imaging is performed to evaluate the atom number. The absorption imaging is based on the comparison between the intensity *I* of a light pulse going through the atoms and the intensity *I*_0_ of a reference light pulse that propagates in the absence of atoms and the Beer’s law, *I*(*x_i_*, *z_j_*) = *I*_0_(*x_i_*, *z_j_*)*e*^−*OD*(*x*_*i*_, *z*_*j*_)^. The optical density (OD) is proportional to the column density of the atoms at a given position ∫*n*(*x*, *y*, *z*)*dy*, where *x* and *z* are the object plane positions corresponding to *x_i_* and *z_j_*, respectively. The number of atoms *N*(*x_i_*, *z_j_*) imaged by the pixel is $N(x_{i},z_{j})=\frac{A}{\sigma_{0}}\mathrm{OD}(x_{i},z_{j})$, where *A* is the pixel area in the object plane, σ_0_ = 3λ^2^/2π is the cross section for resonant atom-light scattering, and λ ≈ 780 nm is the optical transition wavelength. The total atom number is equal to ∫*N*(*x*, *z*)*dxdz*. We can then reliably determine the relation between population transfer and *T*_R_ as presented in Fig. 2A, e.g., 10 μs corresponds to θ = π/2, 20 μs corresponds to θ = π, and 40 μs corresponds to θ = 2π.

In Fig. 5, we explained how the values of θ are obtained from the measurement of population transfer when we apply TR_3_ (*T*_R_ in the main text). Stern-Gerlach splitting was used to separate the *m_F_* = 1 and *m_F_* = 2 parts, and absorption imaging was performed to evaluate the atom number. See the details in the figure caption.

### The CCD image of the interference pattern while θ is scanned

**Fig. 6:**
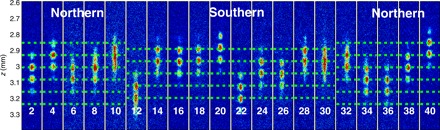
The interference pattern versus *T*_R_ when *T*_G_ = 17 μs (Δϕ ≃ π). The number in each subfigure indicates the duration of *T*_R_ in microseconds. When the Bloch vectors are in the northern hemisphere, the interference phase is seen to be rigid (fixed). When the Bloch vectors cross the equator at *T*_R_ = 10 μs, there is a π phase jump. The interference phase will jump by another π when the vectors cross the equator again at *T*_R_ = 30 μs. Namely, phase rigidity appears when the Bloch vectors are located in either the northern or southern hemisphere, with a π phase jump in between, as presented in Fig. 2 (B to D) and Fig. 3B. The fluctuations in the interference pattern’s location are due to fluctuations in the initial conditions from shot to shot, while the inferred phase is stable, as explained in (*34*).

In Fig. 6, we showed the raw data of the interference patterns, which are displayed in Fig. 2 (averaged over numerous values of θ) and in Fig. 3B (where the phase for different values of θ is presented), when TG_3_ (*T*_G_ in the main text) equals 17 μs (Δϕ ≃ π). The whole scanning range of *T_R_* is 40 μs, corresponding to one full cycle (2π) of the Rabi oscillation. The phase of the interference pattern was found to be rigid when the Bloch vector is located in the northern hemisphere or in the southern hemisphere, with a π phase jump in between.

The interference pattern was fitted with the function *A*exp$\left[-\frac{{\left(z-z_{\text{CM}}\right)}^{2}}{{2\sigma }_{z}^{2}}\right]$$\{1+v\text{sin}[\frac{2\pi }{\lambda }(z-z_{\text{ref}})+\Phi ]\}+c$, where *A* is a constant related the optical density in the system, *z*_CM_ is the center-of-mass (CM) position of the combined wave packet at the time of imaging, σ*_z_* is the Gaussian width of the combined wave packet obtained after time of flight, $\lambda =\frac{\mathrm{ht}}{\mathrm{md}}$ is the fringe periodicity, *v* is the visibility, *z*_ref_ is a fixed reference point, *c* is the background optical density from the absorption imaging, and Φ is the phase of the interference pattern that appears in Eq. 2. In the fringe periodicity $\lambda =\frac{\mathrm{ht}}{\mathrm{md}}$, *h* is the Planck constant, *t* is the duration of time of flight, *m* is the mass of ^87^Rb atom, and *d* is the distance between the two wave packets. In Fig. 3, we measure the dependence of Φ on θ (*T*_R_) for a fixed *T*_G_ and then fit the data to Eq. 2, returning values for both ϕ_0_ and Δϕ.

### Geometrical phases for different values of ∆ϕ

Here, we describe the approach used to derive the expression of Φ*_G_* in Eq. 3. Mukunda and Simon (*37*) developed a general formalism called the quantum kinematic approach for the GP in quantum systems.

In the formalism of Mukunda and Simon, a one-parameter smooth curve was defined from a vector ψ belonging to a Hilbert space $H,C=\{\psi (s)\in N_{0},s\in [s_{1},s_{2}]\}$. $N_{0}$ is the subset of unit vectors of H. Note that the curve C is not necessarily closed. The only requirements of the theory are the smoothness of C, i.e., ψ(*s*) should be differentiable, and the non-orthogonality of the initial and final states. The GP is given by

$$\Phi_{G}=\Phi -\Phi_{D}$$ (4)

where Φ is the total phase. Φ_D_ is the dynamical phase arising from the energy dependence on *s* during the evolution. This general formalism naturally reduces to the evolution under the time-dependent Schrödinger equation if the parameter *s* is time. The curve C is the trajectory of the wave function during the propagation time 0 ≤ *t* ≤ *T*.

The total phase Φ during an evolution along C is given by

$$\Phi =\text{arg}\;\langle \psi (s_{1})|\psi (s_{2})\rangle$$ (5)

Taking $\psi (s)=(\text{cos}\frac{\theta }{2}|2\rangle +\text{exp}\;(\text{is}\Delta \phi )\text{sin}\;\frac{\theta }{2}|1\rangle )$ and {*s*_1_, *s*_2_} = [0,1], we found for the total phase

$$\Phi =\text{arctan}\;\{\frac{{\text{sin}}^{2}(\theta /2)\text{sin}\;\Delta \phi }{{\text{cos}}^{2}(\theta /2)+{\text{sin}}^{2}(\theta /2)\text{cos}\;\Delta \phi }\}$$ (6)

where we should add to Φ the phase ϕ_0_ arising from the evolution of the spatial part. This yields Eq. 2.

The dynamical phase Φ_D_ can be calculated from the integral of the evolution curve C (*7*)

$$\Phi_{D}=\text{Im}\int_{s_{1}}^{s_{2}}\langle \psi (s)|\dot{\psi }(s)\rangle \mathrm{ds}$$ (7)

We find

$$\Phi_{D}=\frac{\Delta \phi }{2}(1-\text{cos}\;\theta )$$ (8)

to which phase ϕ_0_ should also be added. Subtracting Φ_D_ from Φ yields the expression for Φ_G_ of Eq. 3. Φ_G_ is more suitable to use for analysis because gauge-dependent phases in Φ and Φ_D_ mutually cancel.

In fig. S2, we presented the detailed theoretical behavior of Φ_G_ (Eq. 3) as a function of θ and Δϕ. The characteristics of Φ_G_ are the singularity at Δϕ = π and θ = (*n* + 1/2)π (where *n* is an integer), and the change of sign when θ goes across these values. This result was originally obtained from (*10*) (see figure 4 in this reference).

## Supplementary Material

http://advances.sciencemag.org/cgi/content/full/6/9/eaay8345/DC1

Download PDF

An experimental test of the geodesic rule proposition for the noncyclic geometric phase

## SUPPLEMENTARY MATERIALS

Supplementary material for this article is available at http://advances.sciencemag.org/cgi/content/full/6/9/eaay8345/DC1 Fig. S1. Detailed scheme of the spatial SU(2) interferometer. Fig. S2. Theoretical curves of the GP Φ_G_ versus θ for different values of Δϕ.
