# Supplementary material for: An experimental test of the geodesic rule proposition for the noncyclic geometric phase
Source: Sci Adv. 2020 Feb 28;6(9):eaay8345. doi: 10.1126/sciadv.aay8345 (PMC7048419; doi:10.1126/sciadv.aay8345)
Supplement: http://advances.sciencemag.org/cgi/content/full/6/9/eaay8345/DC1 [file supp_6_9_eaay8345__index.html]

Science Advances | Science AdvancesAAASSearchScience AdvancesMenu

## Supplementary Materials

**This PDF file includes:**

- Fig. S1. Detailed scheme of the spatial SU(2) interferometer.
- Fig. S2. Theoretical curves of the GP ΦG versus θ for different values of Δϕ.

Download PDF

**Files in this Data Supplement:**

- Adobe PDF - aay8345\_SM.pdf
