# Supplementary material for: An experimental test of the geodesic rule proposition for the noncyclic geometric phase
Source: Sci Adv. 2020 Feb 28;6(9):eaay8345. doi: 10.1126/sciadv.aay8345 (PMC7048419; doi:10.1126/sciadv.aay8345)
Supplement: Download PDF [file aay8345_SM.pdf]

Science Advances
AAAS

advances.sciencemag.org/cgi/content/full/6/9/eaay8345/DC1

Supplementary Materials for

An experimental test of the geodesic rule proposition for the noncyclic geometric phase

Zhifan Zhou, Yair Margalit, Samuel Moukouri, Yigal Meir*, Ron Folman

*Corresponding author. Email: ymeir@bgu.ac.il

Published 28 February 2020, *Sci. Adv.* **6**, eaay8345 (2020)
DOI: 10.1126/sciadv.aay8345

This PDF file includes:

Fig. S1. Detailed scheme of the spatial SU(2) interferometer.
Fig. S2. Theoretical curves of the GP Φ_G versus θ for different values of $\Delta\phi$.

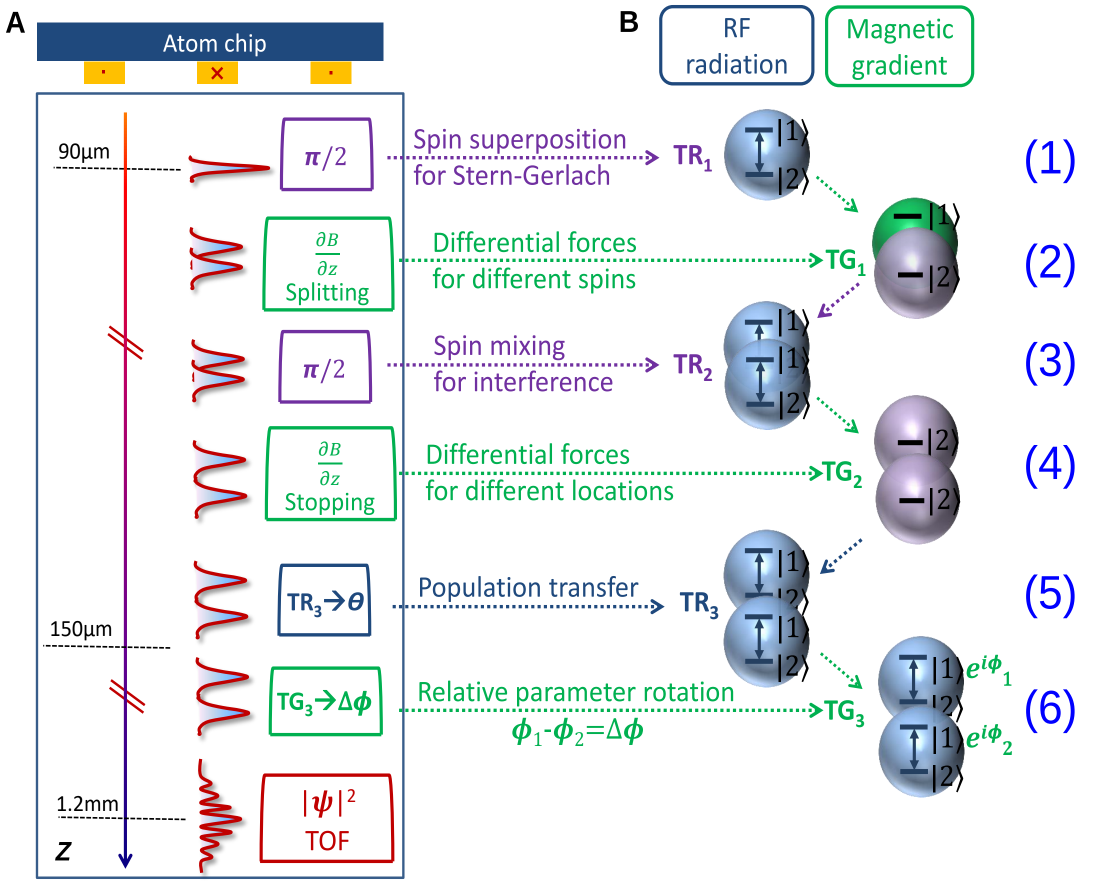

Fig. S1. Detailed scheme of the spatial SU(2) interferometer. **(A)** Detailed sequence (not to scale) of the longitudinal interferometer along the z axis (the direction of gravity). **(B)** The evolution of the states along the sequence. After the atoms are released from the trap, one radio-frequency $\pi/2$ pulse (TR_1) is applied to create an equal superposition of $|2\rangle \equiv |F=2, m_F=2\rangle$ and $|1\rangle \equiv |F=2, m_F=1\rangle$ states (1). These two spin states are then exposed to a differential force created by a magnetic gradient pulse $\partial B/\partial z$ of duration TG_1 (2), generated by currents in the atom chip wires, leading to different accelerations, and, as a result, different positions and different final velocities of the two states. A second $\pi/2$ pulse (TR_2) (3) is applied to mix the spins in each one of the wave packets and then, to stop the relative velocity of the wave packets, a second second magnetic gradient pulse (TG_2) (4) is applied to yield differential forces for the same-spin states which are at different locations. As during TG_2, the $|1\rangle$ state from the two wave packets are pushed outside the experimental zone, the system then consists of two wave packets in the $|2\rangle$ state (separated along the z axis, with zero relative velocity). This 2-level system is initialized with a third RF pulse (5) of duration TR_3 (T_R in the text), after which the relative phase of the two wave packets ($\Delta\phi$) may be changed by applying the third magnetic field gradient (6) of duration TG_3 (T_G in the text). Last, before an image is taken, the wave packets are allowed to expand and overlap.

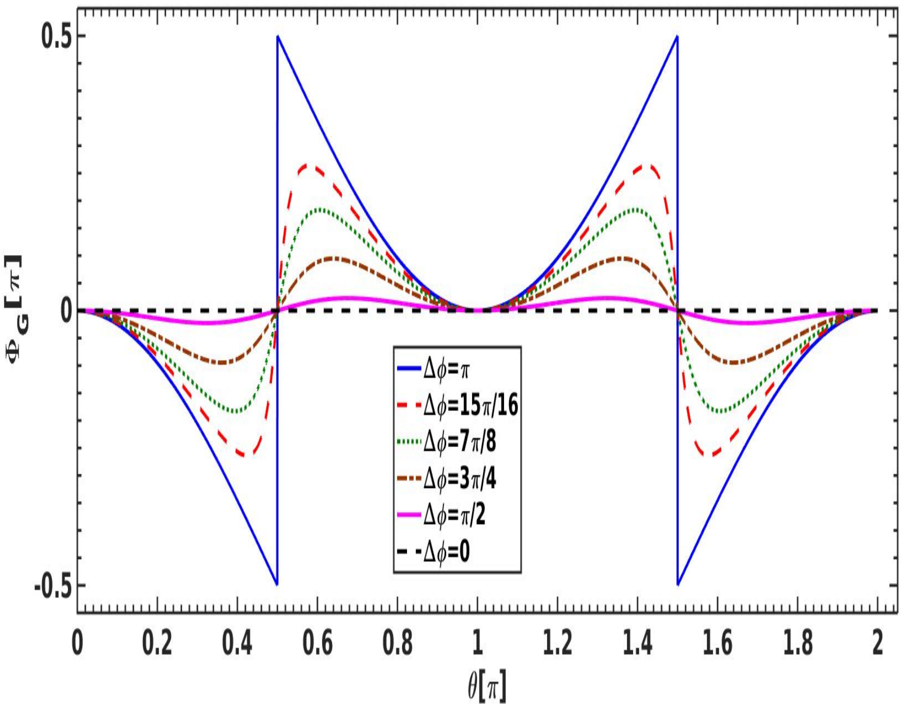

Fig. S2. Theoretical curves of the geometric phase Φ_G versus θ for different values of $\Delta\phi$. Theoretical curves of the geometric phase Φ_G as given by Eq. 3 as a function of θ for different values of $\Delta\phi$. The situation of $\Delta\phi = \pi$ has been presented in Fig. 4B together with the experimental data; the situation of $\Delta\phi = 0.71\pi$ close to $3\pi/4$ has been presented in Fig. 4D together with the experimental data. Both show good agreement between data and theory.
